# Delayed Mesoderm and Erythroid Differentiation of Murine Embryonic Stem Cells in the Absence of the Transcriptional Regulator FUBP1

**DOI:** 10.1155/2017/5762301

**Published:** 2017-05-15

**Authors:** Josephine Wesely, Marlene Steiner, Frank Schnütgen, Manuel Kaulich, Michael A. Rieger, Martin Zörnig

**Affiliations:** ^1^Georg-Speyer-Haus, Institute for Tumor Biology and Experimental Therapy, Paul-Ehrlich-Strasse 42-44, 60596 Frankfurt/Main, Germany; ^2^LOEWE Center for Cell and Gene Therapy Frankfurt and Department for Medicine, Hematology/Oncology, Goethe University Hospital Frankfurt/Main, 60590 Frankfurt/Main, Germany; ^3^Institute of Biochemistry II, Goethe University Frankfurt, 60590 Frankfurt/Main, Germany; ^4^German Cancer Consortium (DKTK), 69120 Heidelberg, Germany

## Abstract

The transcriptional regulator far upstream binding protein 1 (FUBP1) is essential for fetal and adult hematopoietic stem cell (HSC) self-renewal, and the constitutive absence of FUBP1 activity during early development leads to embryonic lethality in homozygous mutant mice. To investigate the role of FUBP1 in murine embryonic stem cells (ESCs) and in particular during differentiation into hematopoietic lineages, we generated *Fubp1* knockout (KO) ESC clones using CRISPR/Cas9 technology. Although FUBP1 is expressed in undifferentiated ESCs and during spontaneous differentiation following aggregation into embryoid bodies (EBs), absence of FUBP1 did not affect ESC maintenance. Interestingly, we observed a delayed differentiation of FUBP1-deficient ESCs into the mesoderm germ layer, as indicated by impaired expression of several mesoderm markers including *Brachyury* at an early time point of ESC differentiation upon aggregation to EBs. Coculture experiments with OP9 cells in the presence of erythropoietin revealed a diminished differentiation capacity of *Fubp1* KO ESCs into the erythroid lineage. Our data showed that FUBP1 is important for the onset of mesoderm differentiation and maturation of hematopoietic progenitor cells into the erythroid lineage, a finding that is supported by the phenotype of FUBP1-deficient mice.

## 1. Introduction

The far upstream element (*FUSE*) binding protein 1 (FUBP1) was identified as a transcriptional regulator that binds to the single-stranded AT-rich FUSE DNA sequence 1.5 kb upstream of the *c-myc* promoter [1]. We and others found FUBP1 to be upregulated in a number of tumor entities, such as hepatocellular carcinoma (HCC), prostate, and colorectal cancer [2–5]. Our studies demonstrated an essential role for FUBP1 in HCC tumorigenesis and established FUBP1 as a pro-proliferative and antiapoptotic oncoprotein [4].

In our recent work, we analyzed the physiological role of FUBP1 in two independent functional *FUBP1* knockout mouse models. In both models, FUBP1 deficiency led to embryonic lethality around day E15.5 and a strong anemic phenotype [6]. The embryos displayed a reduced number of hematopoietic stem cells (HSCs) in the fetal liver, and in contrast to wildtype controls, the remaining FUBP1-deficient HSCs were not able to repopulate the blood lineages in a competitive transplantation experiment. Our studies established FUBP1 as an important regulator of HSC self-renewal. In addition, we noticed that the erythroid lineage in the FUBP1 mutant E15.5 embryos showed a diminished proportion of mature cells, hinting towards an erythroid differentiation defect in the absence of FUBP1 [6].

The essential role of FUBP1 in HSC self-renewal raises the question about the potential role of the protein in other stem cells. Interestingly, the pathohistological analysis of *Fubp1* knockout embryos showed abnormalities during the development of the placenta and of lymphoid tissue and an increased parenchymal cellularity in the brain [7]. Embryonic stem cells (ESCs) are pluripotent cells, that is, they possess an infinite self-renewal potential and can differentiate into cells of all three germ layers (ectoderm, endoderm, and mesoderm) and the germline, ultimately contributing to all lineages of the mature organism [8]. Since the 1980s, mouse ESCs can be isolated from the inner cell mass of blastocysts (most suitable at day E 3.5) and cultivated on feeder cells, which usually consist of replication-deficient fibroblasts. Addition of leukemia inhibitory factor (LIF) to the growth medium can substitute the feeder cells, and ESC lines cultured on gelatin-coated plates in the presence of LIF still maintain their stemness [9, 10]. The recent progress in the ESC research field holds high promise for biomedicine and transplantation medicine as well as for the pharmaceutic developmental research [11, 12]. Discovering novel genes important for specific differentiation decisions led to huge efforts to employ ESCs for cellular therapies [13].

A number of protocols for the differentiation of ESCs into a variety of cell types were established in the last two decades of stem cell research [14–16] (for review of literature describing specifically the in vitro differentiation of ESCs towards the hematopoietic lineage see for example [17, 18]). However, the formation of EBs, which represents the early embryonic development, is a spontaneous germ layer differentiation induced by the absence of LIF and used in almost every differentiation protocol as a first step [19, 20]. The embryonic stem cells undergo a rapid differentiation process during the formation of EBs, and the stem cell markers such as Oct4 and Nanog are downregulated. In parallel, a rapid upregulation of markers for the three germ layers ectoderm, endoderm, and mesoderm occurs [21].

The aim of this study was to analyze the function of FUBP1 in murine embryonic stem cells during spontaneous differentiation upon aggregation to EBs in the absence of LIF. In addition, we wanted to employ the induction of erythropoiesis in ESCs as a suitable cell culture model to complement our in vivo studies on the role of FUBP1 during erythropoiesis in FUBP1-deficient mice [6]. We established *Fubp1* knockout ESC clones with the help of the CRISPR/Cas9 technology [22] and analyzed the consequences of FUBP1 deficiency in ESCs and during EB formation using the stem cell markers Oct4 and Nanog [23–26] and a number of differentiation markers indicative for the mesoderm, ectoderm, and endoderm germ layer cells. Finally, we cocultured the ESCs with OP9 cells [27, 28] to study the direct effect of FUBP1 inactivity for erythroid differentiation.

## 2. Materials and Methods

### 2.1. Cell Lines Used, Embryonic Stem Cell Culture, EB Differentiation, and Erythroid Differentiation

The mouse ESC line E14TG2A [29] was cultured on 0.1% gelatin-coated plates in Glasgow's Minimal Essential Medium (GMEM; *Sigma*), supplemented with 2 mM glutamine (*GIBCO*), 1 mM sodium pyruvate (GIBCO), 1x nonessential amino acids (GIBCO), 10% ES cell-qualified FBS (*Thermo Fisher Scientific*), 10% (*v*/*v*) of a 1 : 1000 dilution of *β*-mercaptoethanol stock solution, and 500–1000 u/ml of leukocyte inhibitory factor (LIF; *Chemicon*). ESCs were aggregated to embryoid bodies by plating 10^4^ cells/ml on bacteria dishes in medium without LIF to induce spontaneous differentiation. For erythroid differentiation of ESCs, OP9 stroma cells (ATCC CRL-2749) were used as a coculture system in the presence of human SCF (100 ng/*μ*l; *Peprotech*) and human EPO (2 U/ml; Roche) as described in [27, 28]. Briefly, OP9 cells were seeded 4 days before adding 1 × 10^4^ ESCs to the confluent OP9 cell layer. At day 5 of the coculture, ESCs were replated in a 1 : 200 dilution on new OP9 cells in the presence of SCF. Additionally, mesoderm (FLK-1) and hemangioblast marker (FLK-1/VE-Cad) were analyzed by flow cytometry. At day 12 of coculturing, hematopoietic cells were identified by using CD45 as a marker. For erythroid differentiation, ESCs were cultured on OP9 cells for 5 days, then SCF and EOP were added (without replating) for additional 5 days, before flow cytometry analysis was performed using CD71 and Ter119 as differentiation markers.

### 2.2. Generation of Fubp1 Knockout and Nontarget Control Clones Using the CRISPR/CAS9 System

The lentiviral CRISPR/Cas9 vector *pLCV2v* [30] was used to introduce the two gRNAs #1 (5′-*caaaaattgggggtgatgc*-3′) and #2 (5′-*agatgccctgcagagagcg*-3′) recognizing the first (#2) and second (#1) murine *Fubp1* exon and one nontarget control (NTC) sequence (5′-*ttccgggctaacaagtcct*-3′). E14TG2A cells were transduced and selected with 2 *μ*g/ml puromycin to establish single-cell clones. Analysis of outgrowing potentially FUBP1-deficient clones was performed by western blot and immunohistochemical staining with anti-FUBP1 antibody.

### 2.3. RNA Preparation, cDNA Synthesis, and Quantitative Real-Time PCR

RNA was prepared using the *RNeasy® Mini Kit* (*Qiagen*) according to the manufacturer's manual. 1 *μ*g of total RNA was reverse transcribed using the *Omniscript® Reverse Transcription Kit* (Qiagen) following the manufacturer's instructions, with additional on-column DNaseI digestion. mRNA expression levels were quantified using a *LightCycler480* (*Roche*) with 96 ­well plates (*4-titude*) and *SYBR® Select Master Mix* (Thermo Fisher Scientific). qPCR reactions were performed in technical duplicates in a total reaction volume of 20 *μ*l. mRNA levels were either normalized to *Gapdh* expression and calculated according to the 2^­ΔΔCt^ method [31] or presented as the relative mRNA expression compared to *Gapdh*.

### 2.4. Western Blot Analysis

FUBP1 expression was detected via immunoblot with an anti­FUBP1 (1 : 1000; clone N-15, *Santa Cruz*), and *β*-actin levels were assessed as a loading control using a goat-derived antiserum (1 : 2000; clone C-11, Santa Cruz). As the secondary antibody, a rabbit anti-goat antibody (1 : 10,000; cat. no. 81–1620; *Invitrogen*) was chosen for detection. Quantification of FUBP1 western blots was performed using the *FUSION Fx* system (*Vilber Lourmat*).

### 2.5. Immunohistochemistry

ESCs were trypsinized, and EBs were dissociated with 500 *μ*l Accutase (*Sigma*). After washing with PBS, 50,000 cells were resuspended in 70 *μ*l PBS and spun onto a polysine adhesion slide (Thermo Fisher Scientific) with a *Cellspin II* centrifuge (*Tharmac*). Staining was performed as follows: EtOH solution (10 min; 100°C), *BondTM* Wash (3 min; RT), Peroxid (10 min; RT), anti-FUBP1 (*abcam 181,111*; 30 min; RT), Polymer (8 min; RT), *BondTM* Wash (4 min; RT), DAB (3,3′-diaminobenzidine; 8 min; RT), Hematoxylin (10 min; RT), and H_2_O (1 min; RT).

### 2.6. Flow Cytometry

EBs were dissociated with 500 *μ*l *Accutase* solution (Sigma). After washing with PBS, the single cells were stained with fixable viability dye *APC-eF780* (*eBioscience*), anti-CD309 (FLK-1/VEGFR2)-PE (*BioLegend*), and anti-mouse CD45.2 PerCP-Cyanine5.5 (eBioscience). Alternatively, cells were fixed, permeabilized, and blocked with mouse IgG before staining with PE-conjugated anti-mouse Brachyury antibody (*R&D Systems*) and corresponding PE-conjugated goat IgG isotype control (R&D Systems). Erythroid cells obtained from ESCs cocultured with OP9 cells were collected from the supernatant, washed with PBS, and stained with fixable viability dye APC-eF780, anti-CD71-APC (eBioscience), and anti-Ter119-PE (eBioscience). Undifferentiated ESCs were trypsinized, washed with PBS, and stained with viability dye APC-eF780, anti-SSEA1-V450 (clone MC480; *BD Biosciences*), and anti-SSEA4-PE (eBioMC-813-70; eBioscience). For cell cycle analysis, EBs were stained with anti-CD309-PE as described above. Subsequently, cells were washed with PBS and permeabilized in 100 *μ*l Cytofix/Cytperm (BD Biosciences). Cells were stained with anti-Ki-67-APC (clone 16A8; BioLegend) overnight. Hoechst 33342 (*ImmunoChemistry Technologies*) was added immediately before FACS analysis. Flow cytometry was performed with a *FACSFortessa* (*Becton Dickinson*), and for data analysis, the *FACS Diva* software (Becton Dickinson) or FlowJo® was used.

### 2.7. Statistical Analysis

Statistical analysis was performed with *GraphPad Prism* software (*GraphPad* Software), applying the two-tailed *t*-test. *p* values < 0.05 were considered statistically significant (*p* < 0.05: ∗, *p* < 0.01: ∗∗, *p* < 0.001: ∗∗∗).

## 3. Results

### 3.1. FUBP1 Is Expressed in ESCs and during EB Differentiation

**Figure 1:**
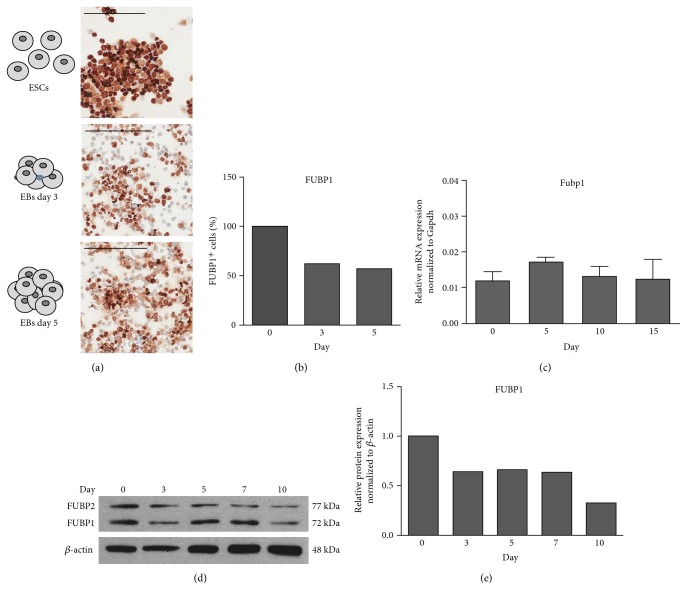
FUBP1 expression in undifferentiated ESCs and during differentiation in EBs. (a) Immunohistochemical (IHC) analysis of ESCs during normal cell culture and dissociated EBs at days 3 and 5 of spontaneous differentiation in the absence of LIF revealed significant but heterogeneous FUBP1 reactivity in the cells. (b) Visual quantification of FUBP1-negative cells based on the IHC analysis shown in (a). Between 267 and 471 cells were counted for each time point, and one slide per time point was analyzed. (c) qPCR analysis of RNA that was isolated from EBs revealed no obvious changes in *FUBP1* mRNA expression during differentiation. (d) FUBP1 protein levels in EBs at different time points of spontaneous differentiation as determined by western blotting. (e) represents the quantification of the blot shown in (d). The qPCR data represent the mean values ± SD (*n* = 3), *C*_t_ values were normalized to *Gapdh* expression. Scale bars in (a) indicate 200 *μ*m.

Undifferentiated ESCs cultured in the presence of LIF and ESCs that were aggregated to EBs for spontaneous differentiation upon plating onto bacterial dishes and removal of LIF were analyzed for the expression of FUBP1. Immunohistochemically (IHC) staining revealed a significant but heterogeneous FUBP1 expression in undifferentiated ESCs on single-cell level (Figure 1(a)). According to our analysis, 38% of all ESCs became FUBP1-negative after 3 days of spontaneous differentiation in EBs (Figure 1(b)). While qPCR experiments (Figure 1(c)) revealed no obvious differences in *Fubp1* mRNA during spontaneous differentiation in EBs, quantification of western blot results (Figures 1(d) and 1(e)) demonstrated a noticeable downregulation of FUBP1 protein levels from day 3 on of EB culture in the absence of LIF, which is in line with the increased amount of FUBP1-negative cells observed by immunohistochemistry (Figures 1(a) and 1(b)).

### 3.2. FUBP1 Expression Is Not Required in Undifferentiated Murine ESCs for Normal Cell Cycle Progression

**Figure 2:**
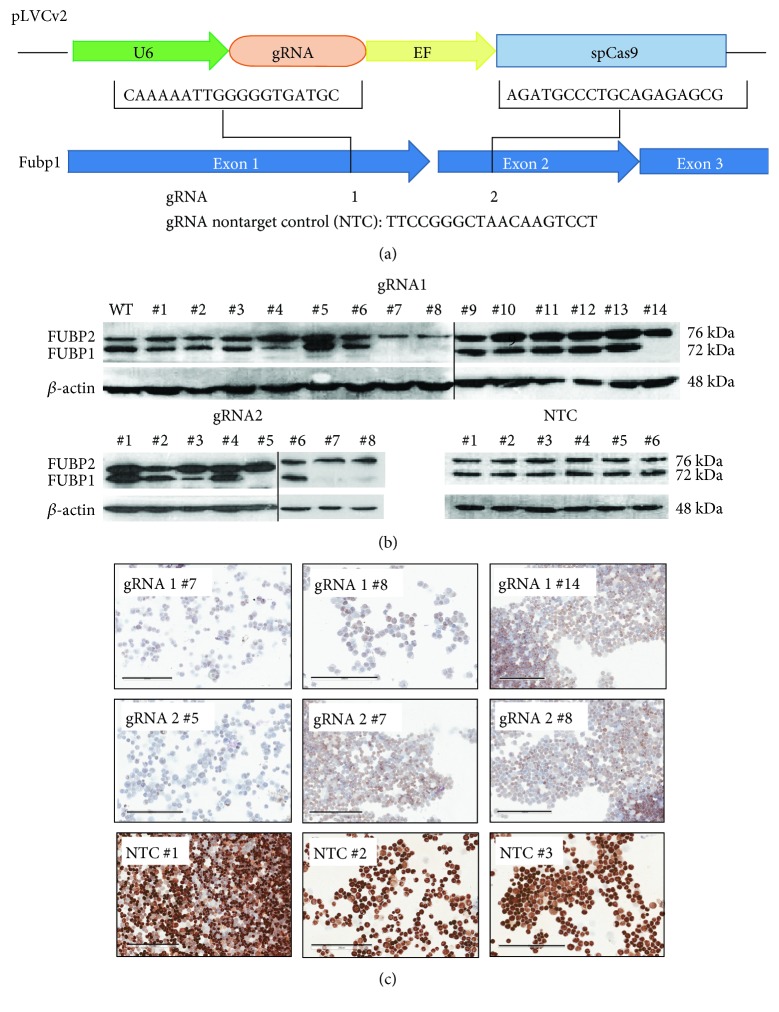
Generation of murine *Fubp1* KO ESC clones. (a) To knockout *Fubp1* in murine ESCs, two gRNA sequences were cloned into the CRISPR/Cas9 plasmid *pLCVv2,* and one nontarget control (NTC) gRNA was cloned as a control. (b, c) FUBP1-deficient ESC clones were identified by western blot analysis (b) and by anti-FUBP1 immunohistochemistry (c) of undifferentiated cells. The weak brownish color of single *Fubp1* knockout cells shown in (c) is most likely due to imperfect washing-off of unbound antibody from parts of the slides with high cell density. Scale bars represent 200 *μ*m.

To investigate the role of FUBP1 in ESC expansion and during differentiation, the lentiviral CRISPR/Cas9 vector *pLCV2v* was used to introduce one of two *Fubp1* gRNAs or a nontarget control sequence (Figure 2(a)). Following puromycin selection of the transduced ESCs, outgrowing clones were isolated and analyzed for FUBP1 protein expression. We could successfully generate 3 *Fubp1* knockout clones each, either transduced with gRNA1 or gRNA2, that showed no FUBP1 expression in western blot analysis (Figure 2(b); gRNA1 #7, #8, #14; gRNA2 #5, #7, and #8; for two of the *Fubp1* knockout clones, the sequence of the targeted locus (exon 2) is shown in Figure S1 in Supplementary Material available online at https://doi.org/10.1155/2017/5762301; the imperfect repair following the CRISPR/Cas9 manipulation led to a change in the reading frame and the generation of premature stop codons in both alleles of both knockout clones). ESC clones that had been transduced with the nontarget control (NTC) gRNA exhibited a comparable FUBP1 protein expression level as detected for untransduced ESCs. Additionally, the 6 *Fubp1* knockout and 3 of the NTC clones were analyzed by immunohistochemistry to confirm the absence of FUBP1 in the knockout clones (Figure 2(c)). No changes in FUBP2 protein expression could be detected in the *Fubp1* knockout or NTC ESC clones. For all further investigations, at least three independent experiments, each with the mean values obtained from several *Fubp1* knockout and NTC ESC clones (as indicated), were performed.

First, we wanted to confirm that both, *Fubp1* knockout and NTC control ESC clones, maintained the same level of pluripotency. Undifferentiated pluripotent mouse ESCs express stage-specific embryonic antigen 1 (SSEA1) which is downregulated during ESC differentiation [32]. In contrast, undifferentiated murine ESCs do not express SSEA4, while their differentiation is accompanied by an increase in SSEA4 expression [33]. Flow cytometry analysis of our ESC clones confirmed their SSEA1^+^/SSEA4^−^ undifferentiated status (see Figure S2), suggesting that the absence of FUBP1 per se does not interfere with murine ESC pluripotency.

The analysis of *Fubp1* knockout and NTC ESC clones showed no significant differences in their cell cycle distribution, and no increase in the number of dead cells could be detected in the absence of FUBP1 (Figure S3A). When we investigated the expression of the two bona fide FUBP1 target genes *p21* and *c-myc* in *Fubp1* knockout and NTC ESC clones, we noticed no difference in *p21* mRNA levels but a significant upregulation of *c-myc* in the *Fubp1* knockout clones (Figure S3B).

### 3.3. FUBP1 Depletion Leads to a Decreased Proportion of Cells Expressing Mesoderm Marker Genes upon Aggregation into EBs

**Figure 3:**
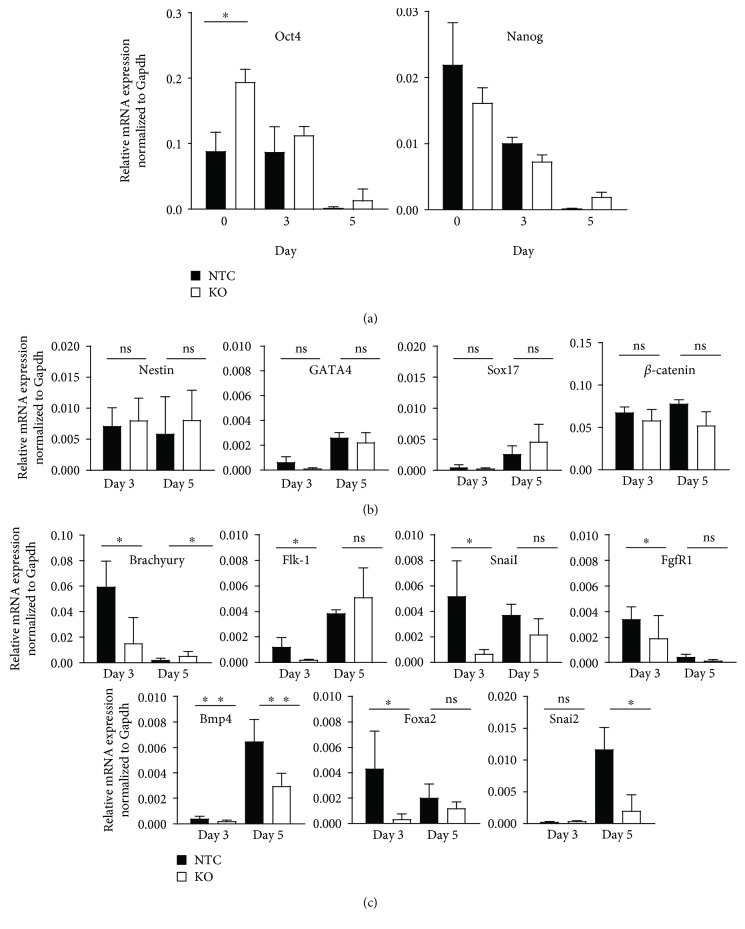
Spontaneous differentiation of wildtype and *Fubp1* knockout ESC clones upon aggregation in EBs. (a) mRNA levels of the stem cell markers *Oct4* and *Nanog* decreased equally in *Fubp1* knockout (KO) and NTC control clones during spontaneous differentiation in EBs. (b) mRNA expression of ectoderm markers (*Nestin and GATA4)* slightly increased during EB formation (day 3 and day 5), but the expression was not affected by the absence of FUBP1. The same was observed for endoderm marker expression (*Sox17* and *β*-catenin) at days 3 and 5. (c) The analysis of the mesoderm markers *Brachyury*, *Flk-1*, *SnaiI*, *FGFR1*, *and Bmp4* revealed significantly reduced mRNA expression levels in *Fubp1* KO clones at day 3 of ESC differentiation. In addition, the Brachyury target gene *Foxa2* was significantly reduced in the *Fubp1* KO compared to NTC control clones at day 3 and the Brachyury target gene *Snai2* at day 5 of EB differentiation. Three independent experiments were performed, each with 4 NTC and 5 *Fubp1* knockout ESC clones. The qPCR data represent the mean values ± SD; data was normalized to *Gapdh* mRNA expression and calculated as relative mRNA expression level (^∗^*p* < 0.05; ^∗∗^*p* < 0.01).

When we aggregated ESCs into EBs in LIF-deficient medium for 5 days to induce spontaneous differentiation, mRNA expression of the stem cell marker genes *Oct4* and *Nanog* decreased as expected in both *Fubp1* knockout and NTC clones (Figure 3(a)). Of note, a significantly increased level of *Oct4* mRNA was observed in the undifferentiated *Fubp1* knockout ESC clones at day 0 of the experiment. Successful differentiation of the ESCs into cells of the three different germ layers was verified using the previously described ectoderm (Nestin and GATA4), endoderm (SOX17 and *β*-catenin), and mesoderm markers (Brachyury, FLK-1, SNAIL, FGFR1, and BMP4). The mRNA expression analysis of the two ectoderm marker genes *Nestin* and *Gata4* and of the two endoderm marker genes *Sox17* and *β-*catenin showed no difference between FUBP1-deficient and NTC ESC clones at day 3 and day 5 of EB differentiation (Figure 3(b)). Surprisingly, when we investigated the mRNA expression of the mesoderm markers at days 3 and 5 of EB differentiation, *Brachyury, Flk-1*, *SnaiI*, *FgfR1*, and *Bmp4* were significantly reduced in the *Fubp1* knockout cells compared to the NTC controls (Figure 3(c)). In line with the decreased *Brachyury* expression in the *Fubp1* knockout cells, mRNA levels of the Brachyury target genes *Foxa2* [34] and *Snai2* [35] were diminished in the FUBP1-deficient differentiating ESCs compared to cells with wildtype FUBP1 expression (Figure 3(c)).

**Figure 4:**
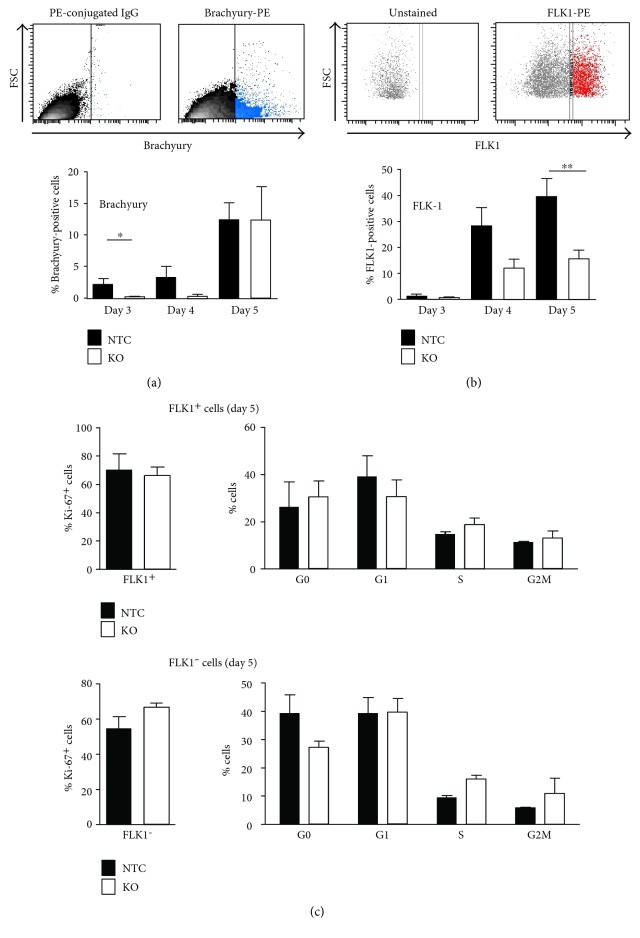
Quantification of the mesoderm marker Brachyury and FLK-1 and cell cycle analysis in cells of differentiating EBs. (a) Intracellular staining of single cells prepared from EB structures for the mesoderm marker Brachyury showed a significant reduction in the proportion of Brachyury^+^*Fubp1* KO cells at day 3 of EB differentiation compared to control cells derived from NTC EBs. This reduction was still visible at day 4, but not any longer at day 5 of EB differentiation, when 12% of *Fubp1* KO and NTC cells derived from EBs were Brachyury^+^. (b) During EB differentiation, the percentage of FLK-1^+^ NTC cells increased from 3% (day 3) to 28% (day 4) and 39% (day 5). In comparison, the proportion of FLK-1^+^*Fubp1* KO cells was clearly decreased. The data represent the mean values ± SD. In each of two independent experiments, 3 (a) or 6 (b) NTC and 3 (a) or 6 (b) *Fubp1* knockout ESC clones were used (^∗^*p* < 0.05; ^∗∗^*p* < 0.01). (c) Quantification of Ki-67^+^ cells in FLK^+^ and FLK^−^ cells in 3 NTC and 5 *Fubp1* KO ESC clones by flow cytometry (left panels). FACS analysis was performed after 5 days of EB aggregation and differentiation in medium without LIF. Right panels: 3 NTC and 5 *Fubp1* KO ESC clones were used for cell cycle analysis of FLK^+^ and FLK^−^ cells following 5 days of differentiation in EBs. For all experiments, data represent the mean values ± SD.

The mRNA expression data were supported by flow cytometry experiments.  Figure 4(a) demonstrates that the amount of intracellularly stained Brachyury^+^ cells at days 3 and 4 of EB differentiation was reduced in *Fubp1* knockout cells compared to NTC clones, while at day 5, the amount of Brachyury^+^ cells was comparable. The quantification of FLK-1^+^ cells by flow cytometry showed a relatively small number of FLK-1-expressing cells at day 3 of EB differentiation (Figure 4(b)). However, at day 4, 30% of all differentiating NTC cells became FLK1-positive, while only 15% of the *Fubp1* knockout cells had started to express FLK1. This difference became even more obvious at day 5 of differentiation (18% FLK^+^*Fubp1* knockout cells versus 40% FLK^+^ NTC cells), supporting the conclusion that FUBP deficiency in ESCs leads to delayed mesoderm differentiation. Additionally, *Fubp1* mRNA levels were slightly increased in a FACS-sorted FLK-1^+^ cell population at day 4 of EB differentiation compared to FLK^−^ cells (Figure S4).

Our previous work had identified a pro-proliferative function of FUBP1 in HSCs [6] and HCC cells [4]. To investigate a potential cell type-specific proliferation defect in differentiating *Fubp1* knockout ESCs that would explain the delayed production of mesoderm cells in the absence of FUBP1, we analyzed the proportion of cycling (Ki-67 positive) FLK1^+^ and FLK1^−^ cells by flow cytometry. In addition, we measured the cell cycle phase distribution of the cells (Figure 4(c)). Overall, we could not detect significant differences in the proliferative behavior of NTC control and *Fubp1* knockout ESC clones, neither in the FLK1^+^ nor in the FLK1^−^ subpopulation of differentiating ESCs.

### 3.4. The Absence of FUBP1 Leads to Diminished Erythroid Differentiation

**Figure 5:**
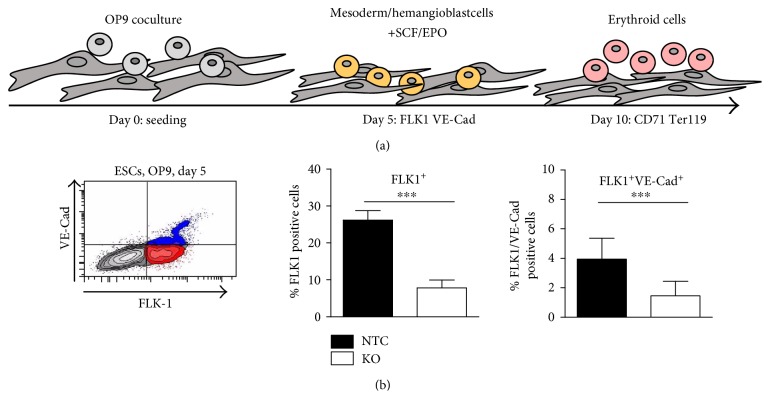
OP9 coculture assay for ESC differentiation into mesoderm/hemangioblast cells. (a) Experimental set-up for OP9 cell-induced ESC differentiation. (b) At day 5 of ESC/OP9 coculture, we analyzed the formation of mesoderm islands and hemangioblast cells and observed a significant reduction in the percentage of FUBP1-deficient FLK1^+^ and FLK-1^+^/VE-Cad^+^ cells compared to NTC control cells. Three independent experiments were performed, each with 3 NTC and 5 *Fubp1* knockout ESC clones. The data represent the mean values ± SD (^∗∗∗^*p* < 0.001).

Homozygous *Fubp1* genetrap mouse embryos lacking FUBP1 activity die in utero with a severe anemic phenotype that is explained by dysfunctional HSC self-renewal [6]. In addition, we had noticed an accumulation of erythroid progenitors during fetal erythropoiesis. The delayed generation of mesoderm cells in the absence of FUBP1 could affect the generation of the hematopoietic lineage. To address this possibility, we used the established coculture assay of ESCs with OP9 cells, which do not produce functional macrophage colony-stimulating factor (M-CSF; [27, 28]), to induce hematopoietic differentiation of ESCs and to analyze the consequences of FUBP1 deficiency for erythropoiesis (see Figure 5(a)). We observed a reduced number of FLK^+^ mesoderm cells and FLK-1^+^/VE-Cad^+^ hemangioblasts in the *Fubp1* knockout cultures compared to the differentiating wildtype ESCs at day 5 of ESC/OP-9 coculture (Figure 5(b)). However, the analysis of CD45^+^ cells at day 12 of OP-9 coculture differentiation (with SCF, but without EPO) resulted in no obvious difference between *Fubp1* knockout and NTC clones (Figure S5).

**Figure 6:**
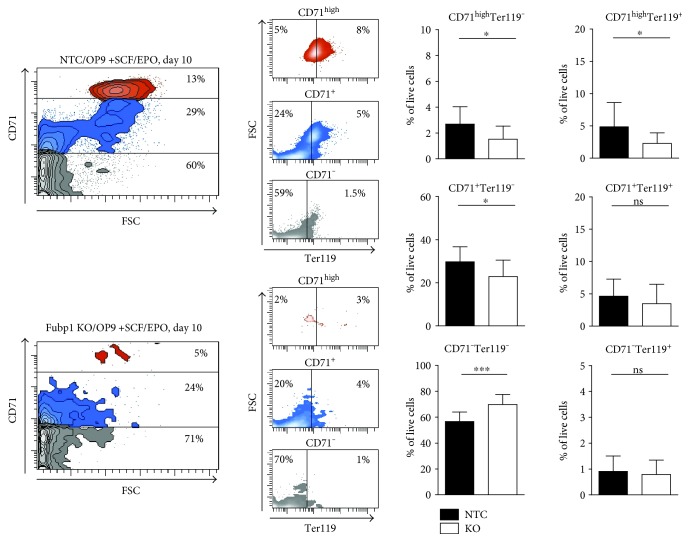
ESC/OP9 coculture in the presence of SCF and EPO for erythroid differentiation. The erythroid differentiation of *Fubp1* KO ESCs was significantly reduced compared to NTC control ESCs, while the amount of CD71^−^/Ter119^−^ ESCs that were not differentiated into the erythroid lineage was increased in the *Fubp1* KO clones (70% KO versus 59% NTC). Maturation of erythroid cells started with the expression of CD71 (CD71^+^/Ter119^−^ cells) in the proerythroblasts. CD71^high^/Ter119^−^ cells: late erythroblasts; CD71^+^/Ter119^+^ cells: reticulocytes; CD71^−^/Ter119^+^: mature red blood cells. Three independent experiments were performed, each with 3 NTC and 6 *Fubp1* knockout ESC clones. The data represent the mean values ± SD (^∗^*p* < 0.05; ^∗∗∗^*p* < 0.001).

Upon specific differentiation into the erythroid lineage by supplementation of the medium with the cytokines stem cell factor (SCF) and erythropoietin (EPO) at day 5 of ESC/OP9 coculture [28], we could generate a high percentage of different maturation stages of the erythroid lineage. Of note, the amount of CD71^−^ Ter119^−^ ESCs that were not differentiated into the erythroid lineage was increased in the *Fubp1* KO compared to the NTC control clones (69.7% versus 56.6%), reflecting a reduced erythroid differentiation of *Fubp1* KO ESCs in the absence of FUBP1 (Figure 6). For the detailed analysis of erythroid marker expression by flow cytometry, we took the morphological changes of the ESCs during erythroid differentiation into account and discriminated first the three CD71^−^, CD71^+^, and CD71^high^ cell populations, before analyzing the Ter119 expression separately for each of these three CD71 subpopulations (Figure 6). Erythroid differentiation started with the expression of CD71 (22.8% CD71^+^/Ter119^−^ KO versus 27.9% CD71^+^/Ter119^−^ NTC cells) in the proerythroblasts. The maturation into early erythroblast cells was indicated by CD71^high^/Ter119^−^ expression (1.4% KO versus 2.7% NTC), and late erythroblasts cells expressed CD71^high^/Ter119^+^ (2.3% KO versus 4.9% NTC). The cells matured further into CD71^+^/Ter119^+^ reticulocytes (3.4% KO versus 4.6% NTC) and finally into mature CD71^−^/Ter119^+^ red blood cells (0.8% KO versus 0.9% NTC).

## 4. Discussion

**Figure 7:**
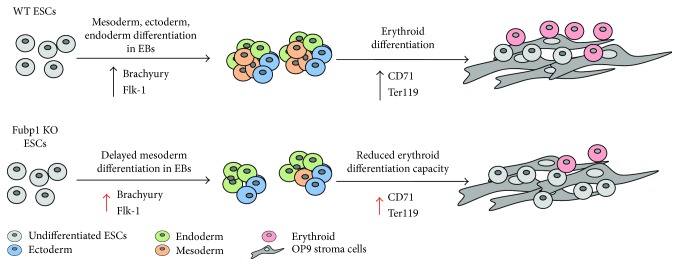
The absence of FUBP1 leads to a delayed mesoderm marker expression during EB formation and a decreased production of mesoderm cells, resulting in a significantly reduced differentiation into erythroid cells.

FUBP1 controls a complex transcriptional network in cells by binding to AT-rich *FUSE* DNA sequences and influencing the transcription of numerous target genes like *c-myc*, *p21, USP29*, and others [36]. Melting of the *FUSE* DNA sequences, that is, the occurrence of single-stranded DNA structures and their recognition by FUBP1, serves as an additional control level to regulate transcription. Most of the FUBP1 literature centers on its role in tumorigenesis, and only recently, an essential physiological role has been described for the protein in HSC self-renewal [6, 7]. In both biological systems, HSCs and FUBP1 (over-) expressing tumor cells, the FUBP1 transcription network provides pro-proliferative and antiapoptotic activity [4, 6]. Based on its prominent stem cell function in HSCs, we now investigated a potential role for FUBP1 in ESCs. We noticed a rather heterogeneous FUBP1 expression level in undifferentiated single ESCs, and during differentiation into the three germ layers in EBs, the overall expression of FUBP1 decreased, probably because a proportion of cells stopped producing FUBP1 protein at all. Surprisingly, removal of FUBP1 expression in murine ESCs using CRISPR/Cas9 technology did not interfere with ESC maintenance and pluripotency (as judged by SSEA1/SSEA4 marker expression). The cell cycle distribution, spontaneous cell death rate, and self-renewal of FUBP1-deficient ESCs did not change compared to NTC control clones. However, upon aggregation of *Fubp1* KO ESCs into EBs and spontaneous differentiation into cells of the three germ layers [19], we observed a significant reduction of mesoderm cell differentiation as shown by the analysis of a variety of mesoderm markers, such as Brachyury and Flk-1 [35, 37, 38]. In contrast, ectoderm and endoderm marker expression was not altered in the absence of FUBP1. As a consequence of the reduced Brachyury levels, the Brachyury target genes *Snai2* [34] and *Foxa2* [35] were significantly reduced in the absence of FUBP1. Although we did not include every germ lineage marker available in our analysis, our data suggest that the accurate enhancement of mesoderm marker expression cannot be implemented at the beginning of EB differentiation in FUBP1-deficient ESCs. This implicates a delayed and insufficient mesoderm cell differentiation, while, at the same time, lack of FUBP1 does not affect proper ESC differentiation into the ectoderm and endoderm lineages in EBs (see Figure 7). Of note, the hematopoietic cell lineages are derived from mesoderm germ layer, and it is well possible that the impaired self-renewal seen in FUBP1-deficient HSCs [6] is connected to our observed defect in mesoderm differentiation. An alternative explanation could be a cell type-specific proliferation defect in FUBP1-deficient differentiating ESCs that would result in an apparent delay of the production of this particular cell type. However, our cell cycle analysis of FUBP1-expressing and FUBP1-deficient FLK1^+^ and FLK1^−^ cells after 5 days of spontaneous differentiation in EBs did not support such FUBP1-dependent differences in the proliferative capacity of particular cell populations.

Mesoderm cells can be further differentiated into hematopoietic cell lineages [39, 40]. In addition to the diminished HSC self-renewal, we had observed an increase in immature erythroid progenitors in *Fubp1* mutant E15.5 embryos, suggesting a differentiation defect in the red blood cell line [6]. The delayed mesoderm differentiation in *Fubp1* knockout ESCs prompted us to differentiate the ESC culture further towards erythropoiesis. In our OP9 coculture system with EPO/SCF supply, we found a higher proportion of less mature FUBP1-deficient cells, resulting in a diminished proportion of further differentiated CD71^+^ Ter119^+^ erythroid cells. Our data support a physiological role of FUBP1 for the differentiation of immature progenitors into functional erythrocytes that extends its HSCs-specific function in hematopoiesis (see Figure 7). Further investigations will aim to identify the relevant target genes within the transcriptional FUBP1 network that are important for timely mesoderm differentiation and proper erythrocyte maturation.

## 5. Conclusion

In the present study, we examined the role of the transcriptional regulator FUBP1 during maintenance and differentiation of ESCs. For this purpose, we generated FUBP1-deficient ESC clones using CRISPR/Cas9 technology. Surprisingly and in contrast to HSCs, FUBP1 seems to be dispensable for ESC self-renewal, and the absence of FUBP1 does not result in reduced proliferation or increased embryonic stem cell death. However, when we aggregated ESCs to EBs for spontaneous differentiation into cells of all three germ layers, we noted a profound delay in mesoderm differentiation from *Fubp1* knockout ESCs. Expression levels of several mesoderm markers, including *Brachyury*, were significantly reduced during differentiation. Cells of the hematopoietic lineage are derived from mesoderm, and in line with the observed mesoderm differentiation defect, we noticed an impaired differentiation capacity of FUBP1-deficient ESCs into the erythroid lineage. Our results substantiate the notion that the proper regulation of the cell type-specific transcriptional network that is controlled by FUBP1 is crucial not only for HSC self-renewal but also for the appropriate differentiation during erythropoiesis.

## Supplementary Material

Supplementary Materials and Methods. Figure S1: CRISPR/Cas9-induced destruction of the Fubp1 open reading frame and creation of premature stop codons. Figure S2: Absence of FUBP1 does not affect ESC pluripotency according to SSEA1/SSEA4 marker expression. Figure S3: Cell cycle and FUBP1 target gene analysis in undifferentiated Fubp1 KO and NTC control ESC clones. Figure S4: Fubp1 mRNA expression in Flk-1+ cells. Figure S5: Differentiation of ESCs into CD45+ hematopoietic cells during OP9 coculture.

## Abbreviations

CRISPR: Clustered regularly interspaced short palindromic repeats
EtOH: Ethanol
EB: Embryoid body
EPO: Eryhropoietin
ESC: Embryonic stem cell
FUBP1: *FUSE* binding protein 1
FUSE: Far upstream element
gRNA: Guide RNA
HSC: Hematopoietic stem cell
IHC: Immunohistochemistry
KO: Knockout
LIF: Leukemia inhibitory factor
M-CSF: Macrophage colony-stimulating factor
NTC: Nontarget control
qPCR: Quantitative polymerase chain reaction
SCF: Stem cell factor.
