# Supplementary material for: Delayed Mesoderm and Erythroid Differentiation of Murine Embryonic Stem Cells in the Absence of the Transcriptional Regulator FUBP1
Source: Stem Cells Int. 2017 May 15;2017:5762301. doi: 10.1155/2017/5762301 (PMC5447289; doi:10.1155/2017/5762301)
Supplement: Supplementary file 1 — Supplementary Materials and Methods. Figure S1: CRISPR/Cas9-induced destruction of the Fubp1 open reading frame and creation of premature stop codons. Figure S2: Absence of FUBP1 does not affect ESC pluripotency according to SSEA1/SSEA4 marker expression. Figure S3: Cell cycle and FUBP1 target gene analysis in undifferentiated Fubp1 KO and NTC control ESC clones. Figure S4: Fubp1 mRNA expression in Flk-1+ cells. Figure S5: Differentiation of ESCs into CD45+ hematopoietic cells during OP9 coculture. [file 5762301.f1.pdf]

SUPPLEMENTARY MATERIAL FOR

Delayed mesoderm and erythroid differentiation of murine embryonic stem cells in the absence of the transcriptional regulator FUBP1

Josephine Wesely, Marlene Steiner, Frank Schnütgen, Manuel Kaulich, Michael Rieger, Martin Zörnig

Supplementary Materials and Methods

qPCR primer (designed for use at T_M 60^0C):

mFubp1 fw	5´-*agaagatggagatcagccagatgc*-3´
mFubp1 rev	5´-*ctttgctgctgatgcattggaggt*-3´
mGapdh fw	5´-*tgtgtccgtcgtggatctga*-3´
mGapdh rev	5´-*ttgctgttgaagtcgcaggag*-3´
mc-myc fw	5´-*cctagtgctgcatgaggagac*-3´
mc-myc rev	5´-*cctcatcttcttgctcttcttca*-3´
mp21 fw	5´-*cctgacagatttctatcactcca*-3´
mp21 rev	5´-*caggcagcgtatatcaggag*-3´
mNanog fw	5´-*gaattctgggaacgcctcatc*-3´
mNanog rev	5´-*ccttgtcagcctcaggacttg*-3´
mOct3/4 fw	5´-*ggacatgaaagccctgcagaa*-3´
mOct3/4 rev	5´-*gacagatggtggtctggctgaa*-3´
mBrachyury fw	5´-*catcggaacagctctccaacctat*-3´
mBrachyury rev	5´-*gtgggctggcgttatgactca*-3´
mNestin fw	5´-*gggccagcactcttagctttgata*-3´
mNestin rev	5´-*tgagccttcagggtgatccag*-3´
mSox17 fw	5´-*ggacacgactgcggagtgaa*-3´
mSox17 rev	5´-*ggtcggcaaccgtcaaatg*-3´
mFlk-1 fw	5´-*gggatggtccttgcatcagaa*-3´
mFlk-1 rev	5´-*actggtagccactggtctggttg*-3´
mFoxa2 fw	5´-*tagcggaggcaagaagacc*-3´
mFoxa2 rev	5´-*cttaggccacctcgcttgt*-3´
mSnail fw	5´-*gccggaagcccaactatagcga*-3´
mSnail rev	5´-*ttcagagcgcccaggctgaggtact*-3´
mSnai2/slug fw	5´-*tggtcaagaaacatttcaacgcc*-3´
mSnai2/slug rev	5´-*ggtgaggatctctggttttggta*-3´
mFgfR1 fw	5´-*gcagagcatcaactggctg*-3´
mFgfR1 rev	5´-*ggagaagtaggtggtatcgctg*-3´
mBmp4 fw	5´-*ccgaatgctgatggtcgttt*-3´
mBmp4 rev	5´-*cctgaatctcggcgactttt*-3´
mß-catenin fw	5´-*tcttcaggacagagccaatgg*-3´
m ß-catenin rev	5´-*accagagtgaaaagaacggtagct*-3´
m Gata4 fw	5´-*caccccaatctcgatatgtttga*-3´
m Gata4 rev	5´-*gcacaggtagtgtcccgtc*-3´

Figure S1: CRISPR/Cas9-induced destruction of the *Fubp1* open reading frame and creation of premature stop codons.

The sequences of the *Fubp1* exon 2 locus for both alleles of two *Fubp1* KO clones revealed a change in the reading frame and the generation of premature stop codons for all 4 alleles. On top of the page, the wildtype *Fubp1* sequence is displayed, and the gRNA 1 target sequence is indicated in bold.

Figure S2: Absence of FUBP1 does not affect ESC pluripotency according to SSEA1/SSEA4 marker expression.

Flow cytometry analysis revealed no difference between FUBP1-deficient and FUBP1-expressing ESC clones in their $SSEA1^{+}/SSEA4^{-}$ status. The results for 2 NTC control and 3 *Fubp1* KO clones are presented, together with the unstained control.

Figure S3: Cell cycle and FUBP1 target gene analysis in undifferentiated *Fubp1* KO and NTC control ESC clones.

A. The cell cycle analysis of propidium iodide-stained cells by flow cytometry showed no differences in cell cycle distribution and the level of apoptotic cell death (subG_1 phase) between *Fubp1* KO and NTC control cells. **B**. The mRNA expression of the direct FUBP1 target genes *p21* and *c-myc* was quantified in *Fubp1* KO and NTC control ESCs. While *p21* expression showed no difference, *c-myc* mRNA was significantly upregulated in *Fubp1* KO clones. Three independent experiments were performed, each with 3 (**A**) and

4 (**B**) NTC and 3 (**A**) and 5 (**B**) *Fubp1* knockout ESC clones. The qPCR data represent the mean values ± SD (**: $p<0.01$).

Figure S4: *Fubp1* mRNA expression in Flk-1$^+$ cells.

After 4 days of EB differentiation, FACS-sorted FLK-1$^+$ showed a slightly higher *Fubp1* mRNA expression compared to FLK-1$^-$ cells. Results were obtained from 3 independent experiments. The data represent the mean values ± SD (**: $p<0.01$).

Figure S5: Differentiation of ESCs into CD45$^+$ hematopoietic cells during OP9 co-culture.

Differentiation of *Fubp1* KO and NTC control ESCs during 12 days of OP9 co-culture plus SCF resulted in the production of CD45$^+$ hematopoietic progenitor cells to a comparable extent. Results were obtained from 3 independent experiments, each with 3 NTC and 5 *Fubp1* knockout ESC clones. The data represent the mean values ± SD.

Fubp1 wildtype sequence

226 bp **gRNA 1 target sequence** 306 bp

-ctgcagagagcgcggcagattgcag**caaaaattgggggtgatgc**tggtacatcattgaattcaaatgactatggttatggg-

-Leu Gln Arg Ala Arg Gln Ile Ala Ala Lys Ile Gly Gly Asp Ala Gly Thr Ser Leu Asn Ser Asn Asp Tyr Gly Tyr Gly -

gRNA 1 #7 (allele 1): deletion of 28 bp

226 bp 278 bp

-ctgcagagagcgcggcag________________________________gtacatcattgaattcaaatgactatggttatggg-

-Leu Gln Arg Ala Arg Gln ________________________________Val His His STOP

gRNA 1 #7 (allele 2): insertion of 1 bp

226 bp 307 bp

-ctgcagagagcgcggcagattgcag**caaaaattgggggtgattgc**tggtacatcattgaattcaaatgactatggttatggg-

- Leu Gln Arg Ala Arg Gln Ile Ala Ala Lys Ile Gly Gly Asp **Cys Trp Tyr Ile Ile Glu Phe Lys STOP**

gRNA 1 #14 (allele 1): insertion of 1 bp

226 bp 307 bp

-ctgcagagagcgcggcagattgcag**caaaaattgggggtgattgc**tggtacatcattgaattcaaatgactatggttatggg-

- Leu Gln Arg Ala Arg Gln Ile Ala Ala Lys Ile Gly Gly Asp **Cys Trp Tyr Ile Ile Glu Phe Lys STOP**

gRNA 1 #14 (allele 2): insertion of 1 bp

226 bp 307 bp

-ctgcagagagcgcggcagattgcag**caaaaattgggggtgattgc**tggtacatcattgaattcaaatgactatggttatggg-

- Leu Gln Arg Ala Arg Gln Ile Ala Ala Lys Ile Gly Gly Asp **Cys Trp Tyr Ile Ile Glu Phe Lys STOP**

Fig. S1

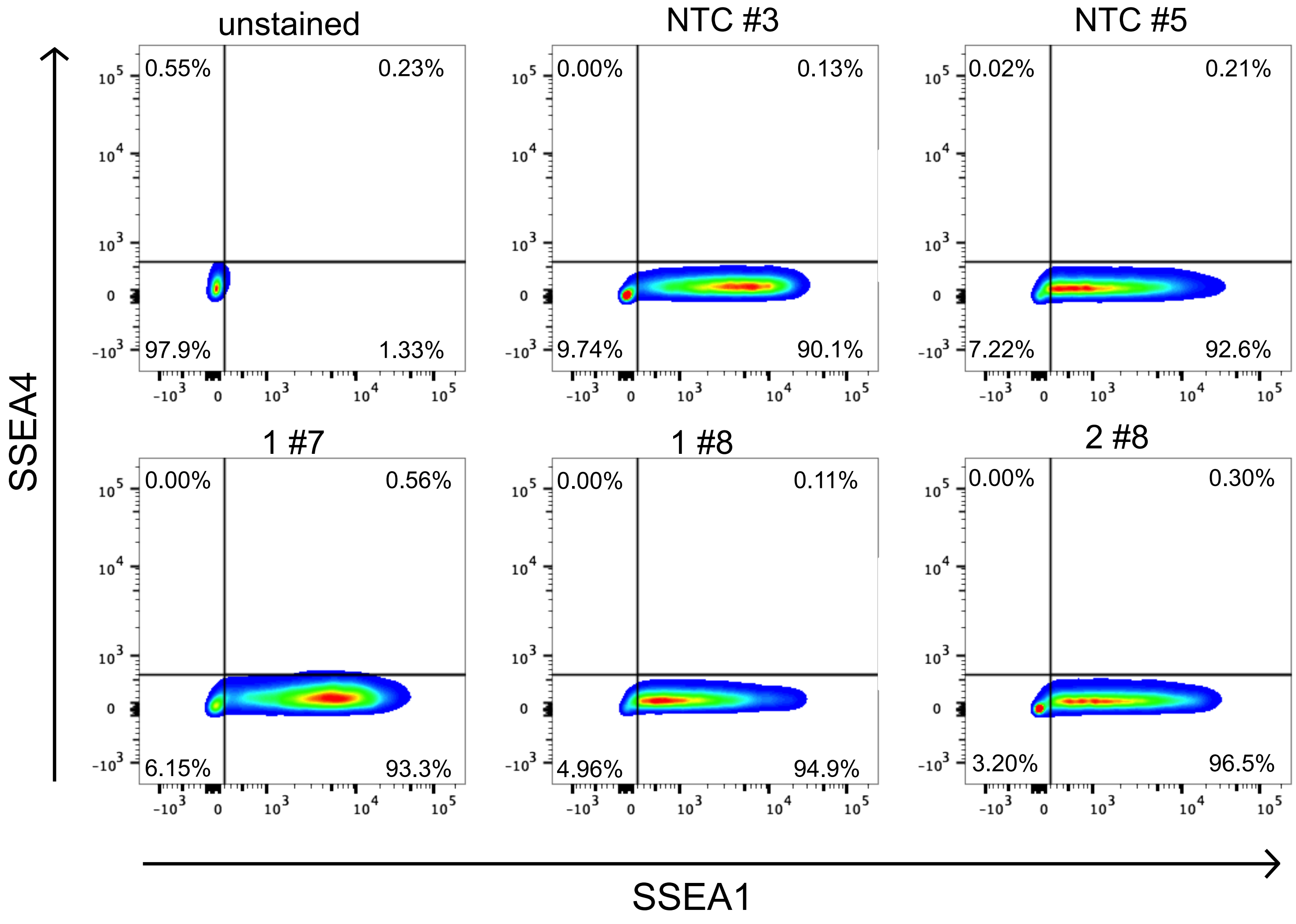
unstained
0.55%
0.23%
97.9%
1.33%
NTC #3
0.00%
0.13%
9.74%
90.1%
NTC #5
0.02%
0.21%
7.22%
92.6%
1 #7
0.00%
0.56%
6.15%
93.3%
1 #8
0.00%
0.11%
4.96%
94.9%
2 #8
0.00%
0.30%
3.20%
96.5%
SSEA4
SSEA1

Fig. S2

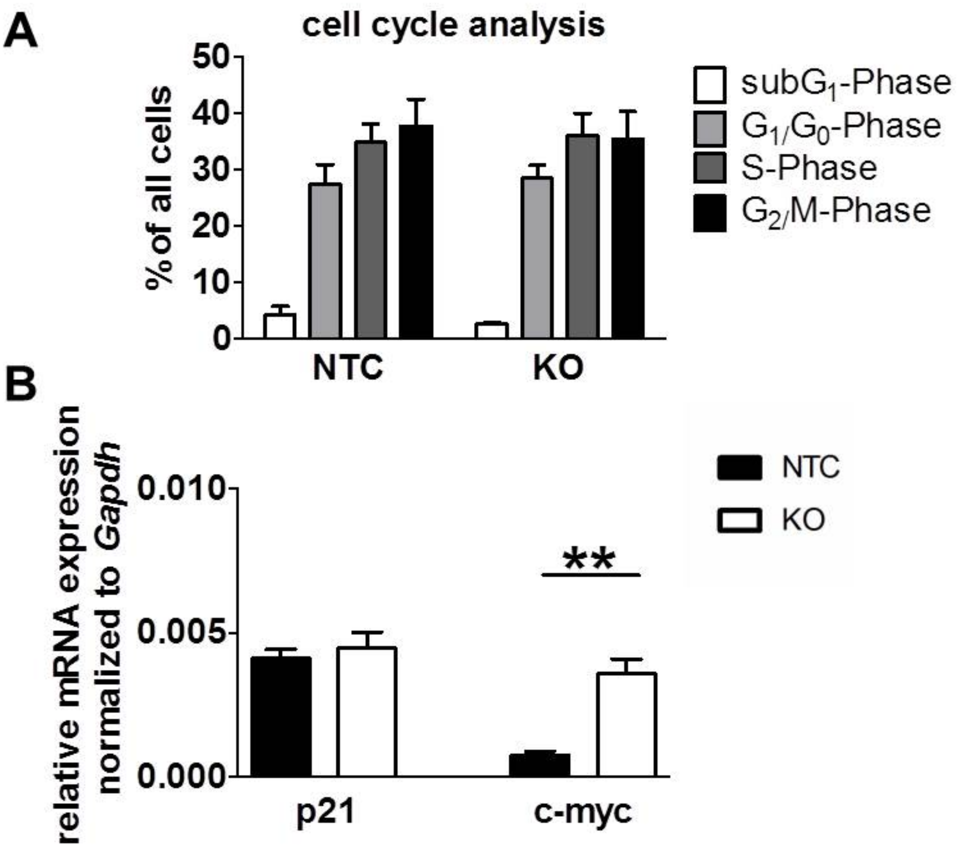
A
cell cycle analysis
% of all cells
50
40
30
20
10
0
NTC
KO
subG1-Phase
G1/G0-Phase
S-Phase
G2/M-Phase
B
relative mRNA expression normalized to Gapdh
0.010
0.005
0.000
p21
c-myc
**
NTC
KO

Fig. S3

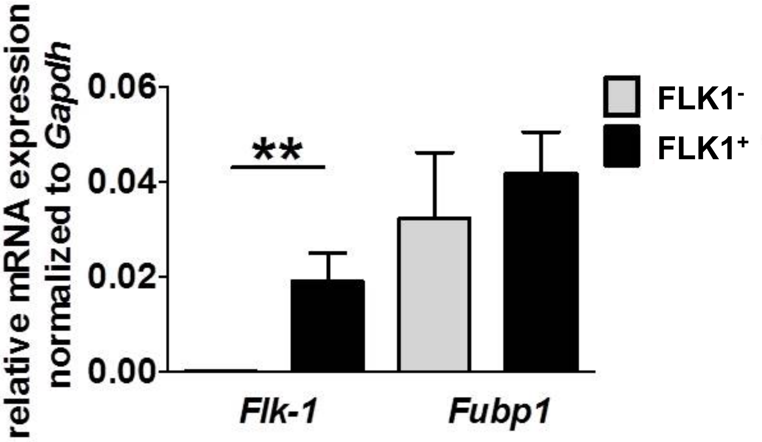
relative mRNA expression
normalized to Gapdh
0.06
0.04
0.02
0.00
**
Flk-1
Fubp1
FLK1-
FLK1+

Fig. S4

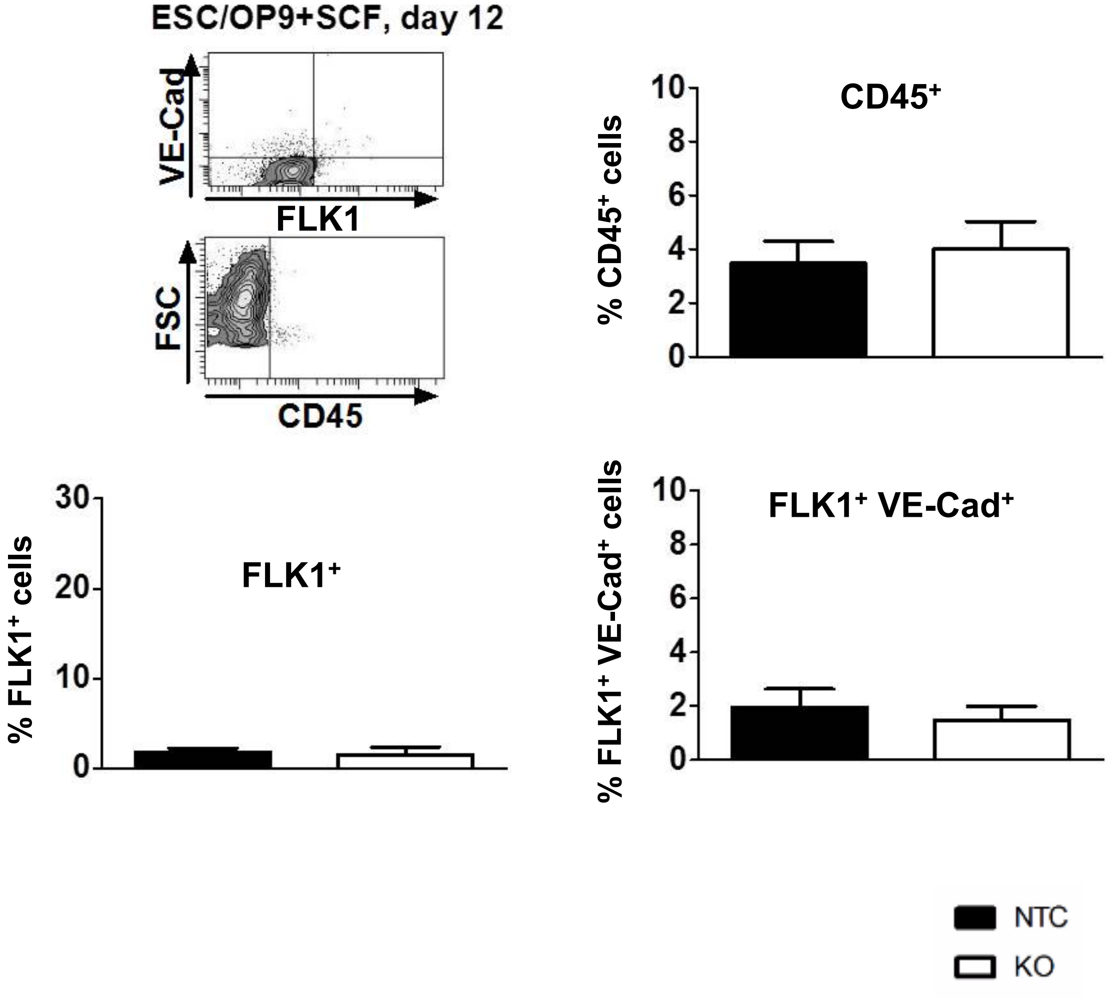
ESC/OP9+SCF, day 12
VE-Cad
FLK1
FSC
CD45
CD45+
% CD45+ cells
FLK1+
% FLK1+ cells
FLK1+ VE-Cad+
% FLK1+ VE-Cad+ cells
NTC
KO

Fig. S5
